# Supplementary material for: Evolution of lateralized gustation in nematodes
Source: eLife. 2025 Jun 30;14:RP103796. doi: 10.7554/eLife.103796 (PMC12208668; doi:10.7554/eLife.103796)
Supplement: Supplementary file 1. [file elife-103796-supp1.docx]

**Table A.** Nematode strains

| **Gene *(Ppa-)*** | **Allele** | **Strain** | **Genetic Lesion or Genotype** |
| --- | --- | --- | --- |
| *--* | *wildtype* | PS312 | *P. pacificus* California reference strain |
| *gcy-22.3* | *csu181* | RLH293 | 2 bp complex deletion causing frameshift and early STOP. 2x outcrossed. |
| *gcy-22.3* | *csu182* | RLH310 | 22 bp complex deletion causing frameshift and early STOP. 2x outcrossed. |
| *gcy-22.3p::GFP* | *csuEx90* | RLH334 | *Ppa-gcy-22.3p::GFP; Ppa-egl-20p::turboRFP* |
| *che-1* | *ot5012* | OH17879 | 4 bp insertion causing frameshift and STOP. 2x outcrossed. |
| *che-1* | *ot5013* | OH17880 | 8 bp complex indel causing frameshift and STOP. 2x outcrossed. |
| *che-1p::HisCl1* | *csuEx83* | RLH336 | *Ppa-che-1pei::optHisCl1; Ppa-egl-20p::turboRFP* |
| *che-1p::RFP* | *lucEx367* | MLC545 | *Ppa-che-1pei::turboRFP; Ppa-egl-20p::turboRFP* |
| *che-1::2xALFA* | *csu226* | RLH325 | *Ppa-che-1::ALFA* (C-terminal tagged to last Exon) |
| *che-1p::RCaMP; che-1p::GFP* | *csuEx93* | RLH335 | *Ppa-che-1pei::optRCaMP; Ppa-che-1pei::optGFP; Ppa-egl-20p::turboRFP* |
| *che-1p::RCaMP;*  *gcy-22.3* | *csu183* | RLH300 | *gcy-22.3(csu181); Ppa-che-1pei::optRCaMP; Ppa-che-1pei::optGFP; Ppa-egl-20p::turboRFP* |
| *ttx-1p::RFP* | *csuEx94* | RLH351 | *Ppa-ttx-1pei::RFP (promoter with first exon and intron)* |
| *ttx-1p::RFP* | *csuEx96* | RLH352 | *Ppa-ttx-1pei::RFP (promoter with first exon and intron)* |
| *ttx-1::2xALFA* | *--* | RLH280 | *Ppa-ttx-1::ALFA* (C-terminal tagged to Exon 18) |
| *--* | *wildtype* | N2 | *C. elegans* Bristol reference strain |

“pei” denotes the regulatory region with promoter, exon 1, and intron 1.

**Table B.** Plasmids

| **Plasmid** | **Reporter** |
| --- | --- |
| *pMM3* | *Ppa-che-1pei::optGFP* |
| *pMM5* | *Ppa-che-1pei::optRCaMP* |
| *pHC30* | *Ppa-che-1pei::optHisCl1* |
| *pVL2* | *Ppa-gcy-22.3p::GFP* |
| *pDC14* | *Ppa-ttx-1pei::RFP* |

**Table C.** Primer sequences

| **Gene (*Ppa-)*** | **Primer** | **Sequence (5’® 3’)** |
| --- | --- | --- |
| *che-1(PPA01143)* | SJC4 | CTACGGTCTTGGGTCGGGCT (crRNA, exon 1) |
| *che-1* | SJC341 | ATCTCTCTCCCTCATCTCCC (forward) |
| *che-1* | SJC342 | TTCCTCCCTCTCCTGACAGC (reverse) |
| *che-1* | SJC343 | AGACGGATGATTAGGGAAGC (forward, nested) |
| *che-1* | SJC344 | GAAGGTGTGGGAGGGTTTCC (reverse, nested) |
| *gcy-22.3 (PPA04454)* | RHL1553 | TCTCACCTTTATGTTCTCTG (crRNA, exon 5) |
| *2xALFA* | ssDNA | TCTAGACTCGAGGAGGAGCTCAGAAGAAGACTCACTGAGtcCCgTctcgaggaggagctcCgTCgTCgTctcacCgag |
| *che-1 (C-terminal ALFA)* | RHL1396 | CACATGAAGACGCACAAGAA (crRNA, last exon) |
| *che-1 (C-terminal ALFA)* | RHL1397 | AGGTCACAAGCCTTATTCGTGC (forward) |
| *che-1 (C-terminal ALFA)* | RHL1399 | GAATCTGTTGCAGCATCTGTTGC (reverse) |
| *gcy-22.3* | RHL1401 | CGGTCCGGCATGCAGTCAAC (forward) |
| *gcy-22.3* | RHL1403 | GTAGTCAGGCAAAGGCTCGA (reverse) |
| *ttx-1 (C-terminal ALFA)* | RHL1510 | CACCGCGTAATCAATACTGC |
| *ttx-1 (C-terminal ALFA)* | RHL1511 | GACCAACTACTATCCTGCAG |
| *ttx-1 (C-terminal ALFA)* | RHL1514 | AGCATGGAGAACTTGGTCCA (crRNA, exon 18) |
